# Effect of birth weight, exclusive breastfeeding and growth in infancy on fat mass and fat free mass indices in early adolescence: an analysis of the Entebbe Mother and Baby Study (EMaBs) cohort

**DOI:** 10.12688/aasopenres.12947.2

**Published:** 2020-01-09

**Authors:** Jonathan Nsamba, Swaib A. Lule, Benigna Namara, Christopher Zziwa, Hellen Akurut, Lawrence Lubyayi, Florence Akello, Josephine Tumusiime, Alison M. Elliott, Emily L. Webb

**Affiliations:** 1 Department of Population Health, London School of Hygiene and Tropical Medicine, London, Keppel Street, WC1E 7HT, UK; 2 Department of Clinical Research, Jeuticals Research and Consulting (U) Ltd, Kampala, Uganda; 3 Department of Infectious Disease Epidemiology, London School of Hygiene and Tropical Medicine, London, WC1E 7HT, UK; 4 Immunomodulation and Vaccines Programme, MRC/UVRI & LSHTM Uganda Research Unit, Entebbe, P.O. Box 49, Entebbe, Uganda, Uganda; 5 Department of Clinical Research, London School of Hygiene and Tropical Medicine, London, WC1E 7HT, UK; 6 Medical Research Council Tropical Epidemiology Group, London School of Hygiene and Tropical Medicine, London, WC1E 7HT, UK

**Keywords:** Birth weight, exclusive breastfeeding, infant growth, fat mass, fat free mass, adolescents, Uganda

## Abstract

**Background:** There is limited data from Africa on the effect of pre- and post-natal growth and infant feeding on later body composition. This study's aim was to investigate the effect of birth weight, exclusive breastfeeding and infant growth on adolescent body composition, using data from a Ugandan birth cohort.

**Methods**: Data was collected prenatally from pregnant women and prospectively from their resulting live offspring. Data on body composition (fat mass index [FMI] and fat free mass index [FFMI]) was collected from 10- and 11-year olds. Linear regression was used to assess the effect of birth weight, exclusive breastfeeding and infant growth on FMI and FFMI, adjusting for confounders.

**Results**: 177 adolescents with a median age of 10.1 years were included in analysis, with mean FMI 2.9 kg/m ^2^ (standard deviation (SD) 1.2), mean FFMI 12.8 kg/m ^2^ (SD 1.4) and mean birth weight 3.2 kg (SD 0.5). 90 (50.9%) were male and 110 (63.2%) were exclusively breastfeeding at six weeks of age. Birth weight was associated with FMI in adolescence (regression coefficient β= 0.66 per kg increase in birth weight, 95% confidence interval (CI) (0.04, 1.29), P=0.02), while exclusive breastfeeding (β= -0.43, 95% CI (-1.06, 0.19), P=0.12), growth 0-6 months (β= 0.24 95% CI (-0.43, 0.92), P=0.48) and growth 6-12 months (β= 0.61, 95% CI (-0.23, 1.46), P=0.11) were not associated with FMI among adolescents. Birth weight (β= 0.91, 95% CI (0.17, 1.65), P=0.01) was associated with FFMI in adolescence. Exclusive breastfeeding (β= 0.17, 95% CI (-0.60, 0.94), P=0.62), growth 0-6 months (β= 0.56, 95% CI (-0.20, 1.33), P= 0.10), and growth 6-12 months (β= -0.02, 95% CI (-1.02, 0.99), P=0.97) were not associated with FFMI.

**Conclusions: **Birth weight predicted body composition parameters in Ugandan early adolescents, however, exclusive breastfeeding at six weeks of age and growth in infancy did not.

## Abbreviations

BMI - Body mass index

CI - Confidence interval

EMaBS- Entebbe Mother and Baby Study

FM - Fat mass

FMI - Fat mass index

FFM - Fat free mass

FFMI - Fat free mass index

NCDs - Non-communicable diseases

SD - Standard deviation

## Introduction

Previously neglected due to high burdens of infectious disease morbidity, attention paid to Non-communicable diseases (NCDs) in Africa has recently increased. Studies suggest that high blood pressure (BP) ^1, 2^ and other cardiovascular diseases (CVDs) ^3^ have escalated on the African continent over recent decades, disproportionately affecting populations at younger ages than in more affluent countries ^4^. The rising burden of NCDs in low and middle-income countries is of public health and economic significance ^5^, given the fragile health care systems and associated cost implications. In Africa, deaths due to NCDs are rising faster than anywhere else in the world ^4^. An understanding of the pathways for development of NCDs in this setting is essential for informing interventions for prevention of NCDs.

Body composition, specifically increased adiposity, is associated with risk of NCDs later in life ^6^ and early-life factors, such as pre- and post-natal growth and infant feeding, have been reported to program and alter body composition ^7^. Sub-optimal nutrition in the fetal or infant periods triggers cellular and epigenetic changes that may affect later body composition ^8^. Rapid growth especially in infancy may result in metabolic changes which can manifest as increased adiposity and result in later NCDs ^9, 10^. Thus, body composition changes might be one of the mechanisms through which early-life exposures may influence susceptibility to NCDs in adulthood.

Evidence, predominantly from high-income countries, has shown that compared to normal birth weight infants, both low and high birth weight infants may bear an increased risk of adulthood obesity ^11^. Rapid weight gain and lack of exclusive breastfeeding in infancy have been associated with increased adiposity in adulthood ^12^. Exclusive breastfeeding has also been reported to be associated with a reduction in fat mass (FM; a measure of adiposity ^13, 14^). However, results are inconsistent, with some studies finding no evidence for the association between birth weight (low or high) and FM ^7, 11, 15, 16^ in late adolescence or adulthood, or for an impact of these early-life factors on risk of NCDs later in life ^17, 18^. Results as reported by some studies ^19^ suggest mixed evidence for an association between birth weight and fat free mass (FFM; a measure of lean muscle mass ^20^) in late adolescence or adulthood.

Few studies from Africa have investigated the relationship between birth weight, exclusive breastfeeding and growth in infancy, and body composition later in life, with tools for measuring body composition not widely available. Studies from South Africa ^21^ and Cameroon ^22^ found that birth weight and linear growth were positively associated with both FM and FFM. However, the impact of early-life factors on later body composition remains understudied among populations from Africa.

## Methods

The current study used prospectively collected data from the Entebbe Mother and Baby Study (EMaBS) birth cohort, conducted in Wakiso district, on the northern shores of Lake Victoria in Uganda. The EMaBS started life as a randomised controlled trial of anthelminthic treatment interventions. A detailed description of the trial design has been given elsewhere ^23^ Briefly, between 2003 and 2005, pregnant women attending antenatal care at Entebbe Hospital and residing in Entebbe Municipality or Katabi sub-county were enrolled into a double-blind randomised placebo-controlled trial designed to evaluate the effect of deworming treatment in pregnancy and childhood on response to childhood vaccines and infections. The trial was completed in 2011 when all children had turned five years of age. After the trial completion, the offspring continued under follow up, being seen at annual routine visits and when sick. Between 20 ^th^ May 2014 and 16 ^th^ June 2016, 10- and 11-year olds in the EMaBS attending the study clinic for their annual visit were enrolled into the EMaBS blood pressure study (BPS). Adolescents participated once in the BPS, on their first 10- or 11- year study visit occurring during the study period. Enrolment into the BPS was postponed for adolescents presenting with malaria (fever with malaria) or other illness until they were free of any illness.

The primary aim of the EMaBS BPS was to investigate whether birth weight and pre- and peri-natal exposures are important in programming BP in children in Uganda; results pertaining to this primary aim are described elsewhere ^24^. From 21 ^st^ January 2015 to 23 ^rd^ December 2015, additional data on body composition (FM and FFM) was collected from EMaBS participants enrolled into the BPS; outside this period the body composition analyser machine was not available. Briefly, adolescents stood barefoot on the posterior electrode base while holding strongly the two anterior electrodes handles of the segmental body composition analyser machine (TANITA BC-418, TANITA Corporation, Tokyo Japan) as described elsewhere ^25^. To avoid ambiguities from using body composition percentages ^26, 27^, height normalized indices (FMI in kg/m ^2^ and FFMI in kg/m ^2^) were computed and used for analysis. FMI is considered as a measure of adiposity and FFMI as a measure of lean muscle mass.

For this analysis, we aimed to investigate if birth weight, exclusive breastfeeding and growth in infancy were associated with body composition (fat mass index [FMI] and fat free mass indices [FFMI]) in early adolescence. Birth weight was measured and recorded to the nearest 0.1 kg for infants delivered in Entebbe hospital using weight scales (Fazzini SRL, Vimodrone, Italy), and captured as recorded on child health cards for infants delivered elsewhere. Further details have been reported previously ^28^. Weight was measured at six months and then annually starting at one year of age using weighing scales (Seca GmbH & Co. KG, Hamburg, Germany). Height was measured at six months and then annually to the nearest 0.1cm using stadiometers (Seca213 GmbH & Co. KG, Hamburg, Germany). Information on feeding practices at six weeks of age was self-reported from the child’s mother or guardian at a six week visit. Data on adolescents’ dietary intake were collected at the time of body composition measurement, by questionnaire.

## Statistical methods

Study exposures were birth weight, breastfeeding status at six weeks, early infant growth (0–6 months) and late infant growth (6–12 months), while the study outcomes were FMI and FFMI at 10 or 11 years of age. Birth weight was considered for analysis as both a continuous variable and as a categorical variable (low birth weight <2.5kg, normal weight 2.5–3.5kg and high birth weight >3.5kg), with analyses run separately for each approach. The 2006 World Health Organisation growth standards ^29^ were used to compute weight for age standardised Z-scores at birth, and at six and 12 months of age. For each participant, growth for the periods 0–6 months and 6–12 months was calculated as the change in Z-score during that period. Growth in each time period (0–6 months, 6–12 months) was categorised as either increased or normal growth using the cut-off of a 0.67 increase in z-score ^10, 30^.

Characteristics of study participants were compared with those of cohort members who did not participate using t-tests and chi-squared tests. Descriptive statistics were calculated as frequencies, means and standard deviations. Spearman’s correlation was used to assess correlations of body composition indices with each other and with birth weight. Linear regression models were fitted separately for FMI and FFMI. Univariable models were first fitted, followed by multivariable models adjusting for confounders. Potential confounders considered were maternal age, body mass index (BMI), education, area of residence and HIV status; household socio-economic index (a score based on building materials, number of rooms and item owned) at enrolment; and offspring’s place of delivery, sex, age at body composition analysis, family history of hypertension, type of school attended, days/week animal-proteins were eaten, days/week fruits were eaten, days/week vegetables were eaten, days/week starchy foods were eaten, days/week sugared drinks were taken. Factors associated with the outcome, or with the exposure of interest were added to the model concurrently and likelihood ratio tests were used to assess adjusted associations between each variable and the outcome.

Current BMI, which can be partitioned into FMI plus FFMI, was considered to be on the causal pathway between birth weight and FMI or FFMI, thus was not considered as a potential confounder for inclusion in regression models. Assumptions underlying the linear regression model analysis (linear relationship between the dependent and predictor variables, homoscedasticity, normally distributed residuals) were investigated using a combination of scatter plots, plots of residuals against fitted values, and normal probability plots. The possibility of multicollinearity due to inclusion of correlated predictor variables was assessed by investigating the change in standard error through calculating variance inflation factors ^31, 32^.

For each of the main exposures, factors associated with that exposure or with the outcome at a 5% level of significance were included in the final model for that exposure. Three *a priori* confounders, household socio-economic status, age and sex were included in the final model regardless of whether associated with the exposure or outcome or not. The test for trend was used to investigate the shape of the relationship between birth weight and the outcomes. Likelihood ratio test p-values were calculated. STATA version 14.2 (College Station, Texas, USA) was used for data analysis. Interaction terms were fitted to assess whether birth weight might modify the effect of breastfeeding or increased growth on the outcomes (FMI or FFMI).

## Ethics and consent

The study was approved by the Research and Ethics Committee of the Uganda Virus Research Institute (GC/127/13/11/35), the Uganda National Council for Science and Technology (MV625) and the London School of Hygiene & Tropical Medicine (Ref:11253). Respectively, written informed consent and assent were obtained from parent/guardian and adolescents for study participation.

## Results

Of the 2345 live born EMaBS offspring, 1119 (47.7%) enrolled into the BPS ^24^ at 10 or 11 years of age, and 177 (7.6%) had data on body composition taken and were included in the analysis. Of the 177 participants included, 90 (50.9%) were male; 175 (98.9%) were singleton births; and 161 (91.0%) were not exposed to maternal HIV in pregnancy ( Table 1, Underlying data ^33^). Regarding the key exposures, the mean birth weight was 3.2 kg (standard deviation (SD) 0.5); 13 (9.4%) had low birth weight, 92 (66.2%) normal birth weight and 34 (24.5%) high birth weight with 38 participants of unknown birth weight. In total, 110 (63.2%) were exclusively breastfed at six weeks of age; with three participants missing data on this exposure. 108 (61%) and 123 (69%) participants had information on growth between 0 and 6 months, and between 6 and 12 months, respectively (the remaining were missing anthropometry for at least one of the time points and thus the change in z-score could not be calculated); 35 (32.4%) had increased growth in the first 6 months of life and 15 (12.2%) had increased growth between 6 and 12 months of age.

**Table 1.** Participant characteristics (N=177).

Characteristics	Frequency/ Mean (sd)	Percentage
**Maternal at enrolment**		
Age, years	24.7 (6.1)	
Household economic index (1 lowest, 6 highest) (n=176)	3.8 (1.1)	
Body mass index (kg/m ^2^)	24.5 (3.3)	
Area of residence (n=176)		
Urban	114	64.8
Rural	62	35.2
Education		
None	4	2.3
Primary	77	43.5
Secondary	76	42.9
Tertiary	20	11.3
HIV status		
Negative	161	91.0
Positive	16	9.0
**Offspring**		
Age, years	10.4 (0.5)	
Birth weight, kg (n=139)	3.2 (0.5)	
Fat mass index	2.9 (1.2)	
Fat free mass index	12.8 (1.4)	
Sex		
Male	90	50.9
Female	87	49.2
Exclusively breastfed at 6 weeks (n=174)		
No	64	36.9
Yes	110	63.2
Place of Delivery		
Entebbe Hospital	127	71.8
Home	20	11.3
Other places	30	17.0
HIV status		
Unexposed	161	91.0
Exposed not infected	14	7.9
Infected	2	1.1
Public hair development (n=174)		
Pre-pubertal	128	73.6
Pubertal	46	26.4
Breast development (girls only) (n=83)		
Pre-pubertal	66	79.5
Pubertal	17	20.5
Days fruit eaten/week (n=174)		
None	13	7.5
1–3	113	64.9
4–7	48	27.6
Days vegetables eaten/week (n=176)		
None	15	8.5
1–3	101	57.4
4–7	60	34.1
Days animal-protein eaten/week (n=176)		
None	14	8.0
1–3	133	75.6
4–7	29	16.5
Days starchy food eaten/week		
1–3	4	2.3
4–7	173	97.7
Days sugared drinks taken/ week (n=176)		
None	63	36.2
1–3	82	46.3
4–7	31	17.5
Type of school attended (n=176)		
Boarding	27	15.3
Day	149	84.7

Percentages may be ± 100 due rounding.

SD; standard deviation.

Missing data: area of residence 1; birth weight 38; pubic hair development 3; breast development 4; days fruit eaten/week 3; days vegetables eaten/week 1; days proteins eaten/week 1; days sugared drinks taken/week 1; type of school attended 1.

Adolescents who had body composition measured were similar to the original EMaBS cohort members who did not participate for most characteristics including maternal (age, parity, BMI, education, place of residence, hypertension, infections [malaria, ascaris, trichuris], trial interventions [praziquantel vs placebo or albendazole vs placebo]) characteristics at enrollment, household socio-economic status at enrollment and childhood (birth weight, sex, feeding status at six weeks of age, HIV exposure status, place of birth, mode of delivery, number of births (twin vs singleton), trial intervention [albendazole]) characteristics, except participants were more likely to be born to separated/divorced/widowed mothers (P-value=0.037) and were less likely to be born to mothers with hookworm infections in pregnancy (P-value=0.036).

At participation, offspring had a median age of 10.1 years (IQR: 10.0 to 10.7), mean BMI 15.8 kg/m ^2^ (SD 1.9), mean FMI 2.9 kg/m ^2^ (SD 1.2) and mean FFMI 12.8 kg/m ^2^ (SD 1.4). Among males, the mean FMI was 2.7 kg/m ^2^ (SD 1.3) and mean FFMI was 13.3 kg/m ^2^ (SD 1.1), while in females the mean FMI was 3.1 kg/m ^2^ (SD 0.9) and mean FFMI was 12.4 kg/m ^2^ (SD 1.5). Birth weight was positively correlated with both FMI (r=0.35, p-value<0.001) and FFMI (r=0.34, p-value<0.001). There was strong correlation between FMI and FFMI with r=0.517, p-value <0.001.

The relationships between the main exposures, and FMI and FFMI are shown in Table 2. Birth weight was analysed separately as a continuous variable and as a categorical variable (the two ways of classifying birth weight were not included in any model together). Unadjusted estimates show that FMI increased by 0.73 kg/m ^2 ^per unit kilogram increase in birth weight, 95% confidence interval (CI):0.33-1.13. When birth weight was treated as a categorical variable, it showed a dose-response relationship with FMI (P-trend=0.007). Further investigation of this dose-response relationship showed no departure from linearity (P=0.92). Exclusive breastfeeding at six weeks (β= -0.19, 95% CI: -0.55, 0.17), increased growth between birth and 6 months of age (β= 0.15, 95% CI: -0.42, 0.71) and increased growth between 6 and 12 months (β= 0.62, 95% CI: -0.10, 1.33) were not associated with FMI in unadjusted analysis. In multivariable analysis birth weight (β= 0.66, 95% CI: 0.04, 1.29) remained associated with FMI; exclusive breastfeeding at six weeks (β= -0.43, 95% CI: -1.06, 0.19), increased growth between birth and 6 months of age (β= 0.24 95% CI: -0.43, 0.92) and increased growth between 6 and 12 months (β= 0.61, 95% CI: -0.23, 1.46) were not associated with FMI.

**Table 2.** Unadjusted and adjusted associations between birth weight, exclusive breastfeeding and growth in infancy, and body composition outcomes (N=177).

Exposures	Unadjusted		Adjusted *	
	β (95 % CI)	p-value	β (95 % CI)	p-value **
** Fat mass index**				
Birth weight (continuous) (n=139)	0.73 (0.33, 1.13)	<0.001	0.66 (0.04, 1.29)	0.019
Birth weight (categorical)				
<2.5 kg (n=13)	Reference		Reference	
2.5 to 3.5 (n=92)	0.54 (-0.18, 1.26)		0.87 (-0.06, 1.80)	
> 3.5 kg (n=34)	1.03 (0.24, 1.82)	0.007 [trend]	1.09 (-0.04, 2.23)	0.051 [trend]
Exclusively breastfed at 6 weeks				
No (n=64)	Reference		Reference	
Yes (n=110)	-0.19 (-0.55, 0.17)	0.538	-0.43 (-1.06, 0.19)	0.122
Growth between 0 to 6 months				
Normal (n=73)	Reference		Reference	
Increased (n=35)	0.15 (-0.42, 0.71)	0.600	0.24(-0.43, 0.92)	0.480
Growth between 6 to 12 months				
Normal (n=108)	Reference		Reference	
Increased (n=15)	0.62 (-0.10, 1.33)	0.089	0.61 (-0.23, 1.46)	0.107
** Fat free mass index**				
Birth weight (continuous) (n=139)	0.68 (0.21, 1.16)	0.005	0.91 (0.17, 1.65)	0.007
Birth weight (categorical)				
> 2.5 kg (n=13)	Reference		Reference	
2.5 to 3.5 (n=92)	0.61 (-0.24, 1.45)		1.11 (0.01, 2.21)	
> 3.5 kg (n=34)	1.16 (0.23, 2.09)	0.009 [trend]	1.53 (0.19, 2.87)	0.020 [trend]
Exclusively breastfed at 6 weeks				
No (n=64)	Reference		Reference	
Yes (n=110)	0.14 (-0.30, 0.57)	0.538	0.17 (-0.60, 0.94)	0.619
Growth between 0 to 6 months				
Normal (n=73)	Reference		reference	
Increased (n=35)	0.36 (-0.29, 1.00)	0.272	0.56 (-0.20, 1.33)	0.100
Growth between 6 to 12 months				
Normal (n=108)	Reference		Reference	
Increased (n=15)	-0.51 (-1.33, 0.32)	0.224	-0.02 (-1.02, 0.99)	0.971

* In multivariable analysis, all factors shown in the table were added to the model together with the exception of birth weight as a continuous variable and birth weight as a categorical variable which were analysed separately (they were not included together in any model). Adjusted associations were adjusted for maternal characteristics at enrolment (household socio-economic status, age, body mass index, HIV status) and adolescents’ characteristics (place of delivery, age, sex, days animal-protein eaten/week, days fruits eaten/week)

** Likelihood ratio test p-value

Birth weight was positively associated with FFMI in unadjusted analysis (β= 0.68, 95% CI: 0.21, 1.16), while exclusive breastfeeding at six weeks (β= 0.14 95% CI: -0.30, 0.57), increased growth between birth and 6 months of age (β= 0.36, 95% CI; -0.29, 1.00) and increased growth between 6 and 12 months (β= -0.51, 95% CI: -1.33, 0.32) were not associated with FFMI. When birth weight was analysed as a categorical variable, findings were consistent with a linear relationship with FFMI (P-trend=0.009, p-value for departure from trend 0.93). In multivariable analysis, birth weight (β= 0.91, 95% CI: 0.17, 1.65) remained associated with FFMI; there remained no evidence of association for the other exposures. There was no evidence that the effect of breastfeeding or growth rate on FMI or FFMI differed by sex or birth weight: for example, for FMI, p-values were 0.97, 0.47 and 0.60 for interaction between birth weight and breastfeeding, growth 0–6 months and growth 6–12 months, respectively. The corresponding interaction p-values for FFMI were 0.12, 0.13 and 0.16, respectively. For all analyses, assessment of the assumptions underlying the linear regression analysis indicated that these were met, and there was no suggestion of multicollinearity.

## Discussion

We hypothesised that birth weight, exclusive breastfeeding and rate of growth in infancy were each associated with body composition indices among Ugandan adolescents aged 10–11 years. This study showed that birth weight was associated with both adolescent FMI and adolescent FFMI but there was no association between exclusive breastfeeding in the first six weeks or growth rate in infancy and FMI or FFMI among early adolescents.

Our findings of a positive association between birth weight and both FMI and FFMI are consistent with results from a cross-sectional study among 557 Cameroonian children aged 5–12 years ^22^, and from a birth cohort study among South Africans, with body composition assessed at ages 10 and 22 years ^21, 34^.

We did not find evidence for an effect of exclusive breastfeeding in the first six weeks on FMI or FFMI. This was contrary to results reported in a meta-analysis ^35^ that showed that on average, each additional month of exclusive breastfeeding reduced adiposity by 4%. The lack of association between exclusive breastfeeding in the first six weeks with adiposity or lean muscle mass development in this study supports results among 18-year-old Brazilians enrolled in a population-based birth cohort ^36^. In our study, only 63% of mothers reported exclusive breastfeeding at six weeks but nearly all mothers [172 (97.2%)] were giving some breast milk and only 2 (1.1%) had weaned, thus a differential effect of breast milk and/or of different feeding patterns may be hard to detect in this population. A limitation of this study is that the relationship between exclusive breastfeeding in the first six months of life, as recommended by WHO, and adolescents’ body composition was not examined because data on feeding status at six months was not collected.

There was no association between increased rate of growth in the first six months of life or from 6 to 12 months and FMI or FFMI. These findings do not support earlier studies predominantly from European counties reviewed in 18, 37 and results from a later study among 909 Dutch term infants ^37^ which reported positive associations between growth rate and body composition. Our study was likely underpowered to detect true associations: of the 177 adolescents for whom body composition data were available, data on growth were only available for around two thirds, thus reducing the sample size for this analysis. Among participants in the larger EMaBS BPS (1119 participants, of which the 177 participants with body composition data were a subset), growth in the first two years of life was positively associated with BP in early adolescence ^24^.

Many studies have used body mass index (BMI) as a surrogate outcome measure for body adiposity. However, evidence to date shows that BMI creates ambiguities since it cannot specifically differentiate between FM and FFM ^38^. We therefore used direct measurement of body composition and the height normalised indices for FM and FFM which are reported to be more precise measures of adiposity and lean muscle, respectively ^26^. The strong correlation between FMI and FFMI suggests that, for the Uganda adolescents participating in our study, FMI and FFMI both increase proportionally with an increase in BMI. This is reflected by the fact that birth weight was positively associated with both increased adiposity and increased lean muscle mass in early adolescence.

We used a segmental bio-electrical impendence body composition analyser to measure body composition among the study adolescents. Bio-electrical impendence has been reported to have good correlation with other methods such as dual energy absorptiometry ^39^ and, importantly in this setting, provided a relatively inexpensive field method of body composition analysis. However, the method relies on prediction equations that are population specific to estimate the parameters of body composition. At the time of the study, there were no validated prediction equations for Uganda’s population.

To our knowledge, this is one of the few studies from East Africa to investigate the impact of early-life factors on the body composition parameters FMI and FFMI. Strengths of the study are its cohort design and the robust methods used for measuring body composition parameters. Data on the exposures of interest and potential confounders were collected prospectively, minimizing recall and reporter bias. Exposures and confounders were determined before the BP study was conceptualized and designed. However, the possibility of residual confounding due to unmeasured variables cannot be ruled out. Some exposure information such as exclusive breastfeeding at six weeks was not available for all of the adolescents. In this study we were unable to differentiate the effects of low birth weight due to growth restriction *in utero* from effects due to pre-term birth because accurate data on gestational age was not available in this population.

Whereas we have investigated the effect of two postnatal factors (rate of growth and exclusive breastfeeding) on later disease risk, further studies should investigate the effect of other postnatal factors such as current diet, age at menarche, sleep patterns/duration and the effect of an obesogenic environment on body composition. In conclusion, exclusive breastfeeding, and infant growth were not associated with body composition among early adolescents from a tropical setting. However, birth weight is a good predictor of both adiposity and lean muscle mass later in life in this setting.

## Data availability

### Underlying data

Figshare: BP_Body_Comp.xlsx. https://doi.org/10.6084/m9.figshare.7775669.v1 ^33^

This project contains the following underlying data:

- BP_Body_Comp.xlsx (Body composition data from the cohort with data dictionary)

### Extended Data

Figshare: Supplementary tables showing primary associations (crude associations between exposure variables and outcomes) https://doi.org/10.6084/m9.figshare.11363048.v1
