# Supplementary material for: Effect of birth weight, exclusive breastfeeding and growth in infancy on fat mass and fat free mass indices in early adolescence: an analysis of the Entebbe Mother and Baby Study (EMaBs) cohort
Source: AAS Open Res. 2020 Jan 9;2:11. Originally published 2019 Mar 14. [Version 2] doi: 10.12688/aasopenres.12947.2 (PMC7118828; doi:10.12688/aasopenres.12947.2)
Supplement: Peer review history

# Peer review: Reviewer response for version 2

**DOI:** 10.21956/aasopenres.14136.r27333

**Published:** 2020-01-10

**Authors:** Tsinuel Girma

**Affiliations:** 1 Department of Pediatrics and Child Health, Jimma University, Jimma, Ethiopia; 2 Department of Global Health and Population, Harvard T. H. Chan School of Public Health, Boston, MA, USA; 3 Department of Nutrition, Exercise and Sports, University of Copenhagen, Copenhagen, Denmark; 4 Public Health, Addis Continental Institute of Public health, Addis Ababa, Ethiopia

Approved. No further comments.

I confirm that I have read this submission and believe that I have an appropriate level of expertise to confirm that it is of an acceptable scientific standard.

# Peer review: Reviewer response for version 1

**DOI:** 10.21956/aasopenres.14024.r26999

**Published:** 2019-08-05

**Authors:** Han C.G. Kemper

**Affiliations:** 1 Amsterdam Public Health, Academic Research Institute, Vrije Universiteit Amsterdam, Amsterdam, The Netherlands

This paper about the Entebbe Mother and Baby Study is well written explaining the important research question about the relationship between birthweight and breastfeeding of the babies with their body composition 10 years later. Fat Mass and Fat Free Mass were used.

 This longitudinal study is very seldom and therefore important to publish. The numbers of subjects is high and the statistical methods to reveal the relationship are up to date. Also the boxplots that are used explain to the scientific reader the results.

 My main question is that the adolescents used in this study differ slightly in calendar age, but between 10 and 12 the biological age can differ largely. So, the authors must take this into consideration. First, maybe they can include data about biological age. Second, there is an anthropometric method to estimate biological age: Mirwald *et al.* (2002 ^1^) published this reliable method. With this the whole analysis can be repeated to see the effects on FM and FMM.

I confirm that I have read this submission and believe that I have an appropriate level of expertise to confirm that it is of an acceptable scientific standard, however I have significant reservations, as outlined above.

## References

[1] : An assessment of maturity from anthropometric measurements. *Medicine & Science in Sports & Exercise*.2002;34(4) : 10.1097/00005768-200204000-00020 689-694 10.1097/00005768-200204000-00020 11932580

# Response

**Published:** 2019-12-11

**Authors:** Jonathan Nsamba

**Affiliations:** London School of Hygiene & Tropical Medicine, Uganda

Thanks so much Han C.G. Kemper for your insightful comments and feedback about the manuscript. The comments have been helpful.  We have outlined our responses in line with each comment made.

 Comment 1:

 My main question is that the adolescents used in this study differ slightly in calendar age, but between 10 and 12 the biological age can differ largely. So, the authors must take this into consideration. First, maybe they can include data about biological age. Second, there is an anthropometric method to estimate biological age: Mirwald et al. (2001) published this reliable method. With this the whole analysis can be repeated to see the effects on FM and FMM.

***Response: Thanks for this comment. The cohort in this analysis was aged between 10 and 12 years, however the age distribution was strongly skewed towards the younger end of this range, with 60% of participants aged between 10 years and 10 years 3 months. Only 35 (20%) of participants were aged 11 years or older. The cohort age structure shows that the study sample were in their early adolescence stage where the factors of biological age might not be as pronounced compared to say 12-year olds and beyond. However, we appreciate the effect that biological age can have on study findings irrespective of calendar age. As reported by Mirwald et al (2001), to calculate maturity/ biological age, we would need data on sitting height and leg length, however, these were not collected. The current study is a secondary data analysis of the already collected data. In future work we would plan to collect data that would allow us to adjust for maturity stage as well as or instead of calendar age.***

# Peer review: Reviewer response for version 1

**DOI:** 10.21956/aasopenres.14024.r26995

**Published:** 2019-07-10

**Authors:** Carlos S. Grijalva-Eternod

**Affiliations:** 1 Institute for Global Health (IGH), University College London, London, UK

This study aimed to generate evidence about the association between birthweight, exclusive breastfeeding and infant growth with body composition at ages 10-11 years in a subsample of 177 Ugandan children that were part of a larger cohort study, the EMaBS study. The study authors collected body composition data using bioelectrical impedance and used the weight data collected at birth, at 6 and 12 months of age to assess prenatal growth (birthweight) and infant growth (a change of weight-for-age z-score between birth and 6 months and between 6 to 12 months greater than 0.67 z-scores).

 The study has the potential to contribute novel findings to the body of literature assessing the early in life contributions to NCD susceptibility later in life from the perspective of the Ugandan context under which the data was collected. I have made a list of comments below that could help the authors improve their work.

**Introduction:**

- Second paragraph: I am unsure if it is correct to state that body composition changes represents a mechanism through which early-life exposure may influence NCDs later in life, given that our current understanding is the early life exposures affects both body composition and NCD susceptibility.
- Third paragraph: Fat mass is incorrectly stated as a proxy of adiposity. Measurement of fat mass is a direct measure of adiposity.

 

**Methods:**

- The methods section would benefit from editing and restructuring. For instance, it might be clearer to describe the three phases that this cohort has undergone in one paragraph, rather than in separate paragraphs. Describe all the measurements used for this analysis in full rather than to direct the reader to a published manuscript (e.g., information about the tools used to assess infant feeding, household wealth, etc.).
- Data handling (e.g. estimating z-scores, defining categories, etc.) and the statistical analysis undertook are clearer when explained separately.
- The description of the analysis does not seem to reflect the information presented in the results section. The methods section mentions the use of correlation analysis, linear regression analysis, likelihood ration tests, etc., but these are not clearly presented in the results.
- Please, provide details about the standard error method mentioned to have been used to assess multicollinearity.
- It is unclear at what age the dietary assessment data used for the analysis was collected.
- The authors do not present a rationale about their choice of methods for assessing infant growth namely, the arithmetic change in z-scores, given the wealth of literature discussing how this selection affects the findings and potentially generate incorrect results (for examples of this discussion see Tu *et al.*, 2006 ^1^ and Lucas *et al.*, 1999 ^2^).

 

**Results:**

- Most of the information provided in the form of tables is replicated in the written narrative. Consider removing it from the narrative to make the manuscript concise and easier to read.
- The value of Figure 1 is unclear, as it is not mentioned within the narrative. Perhaps it would be more informative to present this data in a Hattori chart manner (see Wells, 2000 ^3^).
- Table 2 is confusing. It is unclear what models were tested, whether birthweight was included twice in a model, as a continuous and/or categorical variable, or whether they are presenting separate models. This makes it difficult to assess the authors’ findings.

 

**Discussion:**

- The authors rightly state that they might have been underpowered. It might be useful for readers if they elaborate further of how this might underpin their results.
- The authors should include a discussion about the methods used to assess the variables used for the study. For instance, bioelectrical impedance measures the electrical properties of the body with greater emphasis on lean mass (a good electricity conductor) than of fat mass (a poor electricity conductor). Furthermore, Tanita systems rely for the estimation of lean and fat mass on equations derived on populations different from that of this study. How would this have affected their findings? Would the method used to assess breastfeeding be sensitive enough to differentiate different feeding patterns, what about children that were predominantly breastfed?
- The authors mention segmental body composition, but the relevance of this statement is unclear.
- There is little discussion about the context under which the data was collected, e.g. Ugandan context where HIV is prevalent, and whether this could have or not affected their results.
- Given the sexual dimorphism already observed with body composition data at the ages of 10-11 years, it would be useful for the authors to discuss why they chose to not undertake separate analysis by sex.

I confirm that I have read this submission and believe that I have an appropriate level of expertise to confirm that it is of an acceptable scientific standard, however I have significant reservations, as outlined above.

## References

[1] : Growth, current size and the role of the 'reversal paradox' in the foetal origins of adult disease: an illustration using vector geometry. *Epidemiol Perspect Innov*.2006;3: 10.1186/1742-5573-3-9 9 10.1186/1742-5573-3-9 16884533PMC1557505

[2] : Fetal origins of adult disease-the hypothesis revisited. *BMJ*.1999;319(7204) : 10.1136/bmj.319.7204.245 245-9 10.1136/bmj.319.7204.245 10417093PMC1116334

[3] : A Hattori chart analysis of body mass index in infants and children. *Int J Obes Relat Metab Disord*.2000;24(3) :325-9 1075762610.1038/sj.ijo.0801132

# Response

**Published:** 2019-12-11

**Authors:** Jonathan Nsamba

**Affiliations:** London School of Hygiene & Tropical Medicine, Uganda

Thanks so much Carlos S. Grijalva-Eternod for your insightful comments and feedback about the manuscript. The comments have been helpful.  We have outlined our responses in line with each comment made.

 Comment 1: I am unsure if it is correct to state that body composition changes represents a mechanism through which early-life exposure may influence NCDs later in life, given that our current understanding is the early life exposures affects both body composition and NCD susceptibility.

***Response: Thank you for the comment. We have made a change to the sentence for clarity reasons. It now reads as*** ***“thus, body composition changes *might be** ***one of the mechanisms through which early-life exposures may influence susceptibility to NCDs in adulthood”.***

 Comment 2: Fat mass is incorrectly stated as a proxy of adiposity. Measurement of fat mass is a direct measure of adiposity.

***Response: Thank you for this comment. We have edited the sentence which now reads as “exclusive breastfeeding has also been reported to be associated with a reduction in fat mass (FM; a measure of adiposity).***

 Comment 3: The methods section would benefit from editing and restructuring. For instance, it might be clearer to describe the three phases that this cohort has undergone in one paragraph, rather than in separate paragraphs. Describe all the measurements used for this analysis in full rather than to direct the reader to a published manuscript (e.g., information about the tools used to assess infant feeding, household wealth, etc.).

***Response: Thanks for this comment, our use of paragraphs was meant to point the reader to the cohort phase in which each measurement was done, since the cohort had various phases. We have re-written this section to improve the structure and clarity. We have also included details of the methods used to assess key measures: regarding feeding it now reads “Information on feeding practices at six weeks of age was self-reported from the child’s mother or guardians at a six-week visit.” For household wealth this now reads “household socio-economic index (a score based on building materials, number of rooms and item owned)”.***

 Comment 4: The description of the analysis does not seem to reflect the information presented in the results section. The methods section mentions the use of correlation analysis, linear regression analysis, likelihood ration tests, etc., but these are not clearly presented in the results.

***Response: Thank you for this comment. The correlation results were reported as “Birth weight was positively correlated with both FMI (r=0.35, p-value<0.001) and FFMI (r=0.34, p-value<0.001). There was strong correlation between FMI and FFMI with r=0.517, p-value <0.001”. In addition, table 2 contains the likelihood ratio test p-values that represented the test for trend. Results from regression analysis are presented in table 2 and the supplementary tables which have been added at the end of the manuscript. We have made an addition to the statistical methods to reflect that both univariable (to assess crude associations) and multivariable (to adjust for confounders) linear regression models were run.***

 Comment 5: Please, provide details about the standard error method mentioned to have been used to assess multicollinearity

***Response: Standard errors will be inflated in the presence of multicollinearity as described in ***(Belsley, Kuh & Welsch, 2013; Daoud, 2017) ***. One method of assessing this formally is to calculate variance inflation factors which indicate by how much the standard error increases in the presence of multicollinearity (with a variance inflation factor of 1 indicating no multicollinearity issues). We have expanded the text and included references.***

 Comment 6: It is unclear at what age the dietary assessment data used for the analysis was collected.

***Response: Thanks a lot for this comment. We have amended to manuscript that breastfeeding data was collected at 6 weeks of age and adolescents’ dietary assessment data were collected at the time of the blood pressure study.***

 Comment 7: The authors do not present a rationale about their choice of methods for assessing infant growth namely, the arithmetic change in z-scores, given the wealth of literature discussing how this selection affects the findings and potentially generate incorrect results (for examples of this discussion see Tu et al., 20061 and Lucas et al., 19992).

***Response: Thank you for your comment. We appreciate the risk of misinterpretation of results based on the fetal origin of disease especially if adjustment in the models is only made for current size. For comparison purposes, we used a 0.67 cut off reported by Freeman (1995) generated from a healthy and standard UK population. This represented the rate of rapid growth.***

 Comment 8: The value of Figure 1 is unclear, as it is not mentioned within the narrative. Perhaps it would be more informative to present this data in a Hattori chart manner

***Response: Thanks for this comment: We*** ***appreciate the reviewer’s point and have removed Figure 1*.**

 Comment 9: Table 2 is confusing. It is unclear what models were tested, whether birthweight was included twice in a model, as a continuous and/or categorical variable, or whether they are presenting separate models. This makes it difficult to assess the authors’ findings.

***Response: Thanks for this comment. We have added further clarification on this in both the methods and results sections, and have also added a footnote to the table which reads “*** ***In multivariable analysis, all factors shown in the table were added to the model together with the exception of birth weight as a continuous variable and birth weight as a categorical variable which were analysed separately (they were not included together in any model).”***

 Comment 10: The authors should include a discussion about the methods used to assess the variables used for the study. For instance, bioelectrical impedance measures the electrical properties of the body with greater emphasis on lean mass (a good electricity conductor) than of fat mass (a poor electricity conductor). Furthermore, Tanita systems rely for the estimation of lean and fat mass on equations derived on populations different from that of this study. How would this have affected their findings? Would the method used to assess breastfeeding be sensitive enough to differentiate different feeding patterns, what about children that were predominantly breastfed?

***Response: It is true that prediction equations are population specific, but unfortunately at the time of the study there were no equations validated for Ugandan population. A recent paper by Ndagire (2018) has published prediction equations specifically for Ugandan populations. We have amended the manuscript to highlight this limitation. It now reads as “However, the method relies on prediction equations that are population specific to estimate the parameters of body composition. At the time of the study, there were no validated prediction equations for Uganda’s population.” We have also expanded our discussion of the findings related to breastfeeding to reflect the fact that due to the way the data on this were collected, we were not able to examine the effects of different feeding patterns or of longer term breastfeeding behaviour.***

 Comment 11: The authors mention segmental body composition, but the relevance of this statement is unclear.

***Response: We used a segmental body composition analyser for the estimation of body composition as reflected in the methods section. We however, have edited this section of the discussion and removed the word “segmental” because we did not present body composition data by the cylindrical segment of the body from which it was measured. It now reads as “We used a segmental bio-electrical impendence body composition analyser to measure body composition among the study adolescents.***

 Comment 12: There is little discussion about the context under which the data was collected, e.g. Ugandan context where HIV is prevalent, and whether this could have or not affected their results.

***Response: Thanks for the comment. We considered the role of HIV and other infectious diseases in our study. We collected data for maternal HIV status and the HIV exposure status of the offspring. In our cohort, 16 mothers tested positive for HIV during pregnancy with only 2 offspring becoming infected. Due to these small numbers, we did not include results relating to HIV in our manuscript. We also found a low prevalence of helminths and malaria in our study participants.***

 Comment 13: Given the sexual dimorphism already observed with body composition data at the ages of 10-11 years, it would be useful for the authors to discuss why they chose to not undertake separate analysis by sex.

***Response: Thanks for this comment, we investigated this question and as reported in the manuscript (last paragraph of results section), we found that “*** **There was no evidence that the effect of breastfeeding or growth rate on FMI or FFMI differed by sex or birth weight *”. In light of this finding, we do not present results separately by sex.***

**References**

 Ndagire, C. T., Muyonga, J. H., Odur, B., & Nakimbugwe, D. (2018). Prediction equations for body composition of children and adolescents aged 8-19 years in Uganda using deuterium dilution as the reference technique. Clinical Nutrition ESPEN, 28, 103–109. https://doi.org/10.1016/j.clnesp.2018.09.004

# Peer review: Reviewer response for version 1

**DOI:** 10.21956/aasopenres.14024.r26838

**Published:** 2019-04-24

**Authors:** Tsinuel Girma

**Affiliations:** 1 Department of Pediatrics and Child Health, Jimma University, Jimma, Ethiopia; 2 Department of Global Health and Population, Harvard T. H. Chan School of Public Health, Boston, MA, USA; 3 Department of Nutrition, Exercise and Sports, University of Copenhagen, Copenhagen, Denmark; 4 Public Health, Addis Continental Institute of Public health, Addis Ababa, Ethiopia

The work is original and investigated the relationship between birth weight, exclusive breastfeeding and growth in infancy, and body composition among 10-11 year-old children (early adolescence) from Uganda. The findings will contribute to the understanding of the link between early like exposure and growth with later health and development particularly in populations living in contexts similar with Uganda.

- The study participants were drawn from a birth cohort that was established for a different study. Originally, they had a birth cohort of 2345 live births of which 1119 (47.7%) were enrolled for another study at 10 or 11 years of age. This study used 177 (7.6%) of these who had body composition data. Although the investigators tried to show that the huge drop-out had little selection bias, it was not clear on what variables were they matching or similar (page 4). I suggest to depict the sampling process including exclusion criteria using a PRISMA flow diagram.  
- The data represented mainly urban (65%) and women populations with high HIV prevalence (9%); of which the offspring, 8% were exposed but uninfected while 1% were infected. The data has also included few variables as indicators of stages of puberty. However, it was not clear why the HIV and these variables were not included in the final regression model - see table 2.
- I don't see the purpose of Figure 1. The difference between FMI and FFMI in both sexes is obvious. One option is to show BC by sex. 
- The investigators claim that this study is unique in East Africa (page 8); this does not seem justified. In the broader sense of East Africa, for instance, there are published articles from the Infant Anthropometry and Body Composition (iABC) cohort in Ethiopia that could have been used as reference(s) in this paper.

I confirm that I have read this submission and believe that I have an appropriate level of expertise to confirm that it is of an acceptable scientific standard.

# Response

**Published:** 2019-11-11

**Authors:** Jonathan Nsamba

**Affiliations:** London School of Hygiene & Tropical Medicine, Uganda

Thanks so much Dr. Tsinuel Girma for the insightful comments and feedback about the manuscript. The comments have been helpful.  We have outlined our responses in line with each comment made.

**Comment 1:**

The study participants were drawn from a birth cohort that was established for a different study. Originally, they had a birth cohort of 2345 live births of which 1119 (47.7%) were enrolled for another study at 10 or 11 years of age. This study used 177 (7.6%) of these who had body composition data. Although the investigators tried to show that the huge drop-out had little selection bias, it was not clear on what variables were they matching or similar (page 4). I suggest to depict the sampling process including exclusion criteria using a PRISMA flow diagram. 

***Response:*** *Thanks for this comment. We tried to explicitly explain the sampling flow in words. The PRISMA (Preferred Reporting Items for Systematic Reviews and Meta- Analyses) flow diagram (which depicts the flow of information through the different phases of a systematic review) is not the most appropriate in this situation. We think that this reporting is well suited for this article.*

*We have amended the manuscripts to include the list of variables that were compared for EMaBS participants included and not included in this study. It now reads as *“ *...... for most characteristics including maternal (age, parity, BMI, education, place of residence, hypertension, infections [malaria, ascaris, trichuris], trial interventions [praziquantel vs placebo or albendazole vs placebo]) characteristics at enrollment, household socio-economic status at enrollment and childhood (birth weight, sex, feeding status at six weeks of age, HIV exposure status, place of birth, mode of delivery, number of births (twin vs singleton), trial intervention [albendazole]) characteristics, except participants were more likely to be born to separated/divorced/widowed mothers.....”* *.*

**Comment 2:**

The data represented mainly urban (65%) and women populations with high HIV prevalence (9%); of which the offspring, 8% were exposed but uninfected while 1% were infected. The data has also included few variables as indicators of stages of puberty. However, it was not clear why the HIV and these variables were not included in the final regression model - see table 2.

***Response:*** *Thanks so much for the comment. For each of the main exposures, factors crudely associated with that exposure or with the outcome at a 5% level of significance were included in the final model for that exposure. HIV was not included basing on these criteria. However, a priori confounders, household socio-economic status, age and sex were included in the model regardless of whether associated with the exposure or outcome or not.*

**Comment 3:**

I don't see the purpose of Figure 1. The difference between FMI and FFMI in both sexes is obvious. One option is to show BC by sex. 

***Response:*** *Thanks so much for the comment. However, it is not clear to us. We definitely showed BC by sex.*

**Comment 4:**

The investigators claim that this study is unique in East Africa (page 8); this does not seem justified. In the broader sense of East Africa, for instance, there are published articles from the Infant Anthropometry and Body Composition (iABC) cohort in Ethiopia that could have been used as reference(s) in this paper.

***Responses:*** *Thanks for this comment and the reference. We must say, the Ethiopian study (Abera et al. 2018) should have been used as a reference; we just did not come across it during our literature review and submission of earlier manuscripts (February 2018) since the paper has just been recently published (June 2018). We have amended the sentence in the article to reflect that our study is one of the few studies in East Africa to investigate these relationships. Now reads “To our knowledge, this is one of the few studies from East Africa to investigate the impact of early-life factors on the body composition parameters FMI and FFMI.* *”*
